# Optimal Material Search for Infrared Markers under Non-Heating and Heating Conditions

**DOI:** 10.3390/s21196527

**Published:** 2021-09-29

**Authors:** Yuki Kubota, Yushan Ke, Tomohiko Hayakawa, Yushi Moko, Masatoshi Ishikawa

**Affiliations:** 1 Graduate School of Information Science and Technology, The University of Tokyo, Hongo 7-3-1, Bunkyo-ku, Tokyo 113-8656, Japan; 2 Information Technology Center, The University of Tokyo, Hongo 7-3-1, Bunkyo-ku, Tokyo 113-8656, Japan; ke@ishikawa-vision.org (Y.K.); hayakawa@ishikawa-vision.org (T.H.); moko@ishikawa-vision.org (Y.M.); ishikawa@ishikawa-vision.org (M.I.)

**Keywords:** infrared markers, heat sources, specular reflection, infrared imaging

## Abstract

Research on optimal markers for infrared imaging and differences in their characteristics in the presence of heat sources has not yet been performed. This study investigates optimal material combinations for developing an accurate and detachable infrared marker for multiple conditions in the medium wave infrared (MWIR) region. Based on four requirements, 11 material combinations are systematically evaluated. Consequently, the optimal marker differs in relation to the presence of specular reflection components. Metal–insulator markers are suitable under non-heating and hot-air heating conditions without reflection components, although a printed marker made of copier paper is captured more clearly than metal–insulator markers during heating, using an optical radiation heating source with reflection components. Our findings can be applied in structural health monitoring and multi-modal projection involving heat sources.

## 1. Introduction

Infrared imaging with a thermography camera has been widely utilized for various applications, including multi-modal imaging and structural health monitoring [1,2,3,4]. For these applications, infrared markers play an essential role in device calibration and evaluation. In multi-modal imaging, both infrared and visible cameras capture the observation target, and the markers are mainly used to calibrate the spatial positions of the two cameras [5,6]. For infrastructure inspection, a thermography camera can capture invisible structural deformations, such as floating and peeling. The markers are necessary to evaluate the resolution of the images obtained by the inspection systems in addition to infrared camera calibration [3,7,8,9].

To develop the infrared markers, note that the infrared and visible cameras have different wavelength sensitivities [1,10]. Typically, a simple marker printed on copier paper cannot be captured using an infrared camera. Hence, several marker designs have been proposed for calibration. Using a thermal lamp [11,12], the difference in the heat absorption coefficient between white and black surfaces was ascertained. Thus, although it works with copier paper, this marker could only be used under heating conditions [1]. Markers that can be used without heating sources have been developed using metal–insulator boards [6,13,14,15], wire grids [5,16], and aluminum or wood plates with open slits [17,18]. These markers take advantage of the differences in reflectivity in the infrared region between insulators, metals, and voids. It is also possible to construct markers using a device that converts electricity into heat, including electrical resistors [19], light bulbs [20,21,22], and small circular thermostatic heaters [23].

In addition to the development of markers, several studies have compared the characteristics of materials and markers [24,25]. St-Laurent et al. evaluated the robustness of image clearances after heating using an aluminum board coated with ink to select suitable materials for the calibration board [5]. Saponaro et al. quantitatively evaluated the performance of the printed calibration board, investigating the time scale of the contrast decrease after heating [26]. However, materials suitable for markers need to be investigated for a wider range, and no requirements for developed markers have yet been explicitly proposed. Moreover, the changes to marker performance based on heat sources and marker selection during and after heating have not been investigated. The identification of an optimal combination of marker materials is necessary for efficient monitoring and imaging using an infrared camera. In particular, considering the situation of the inspection and imaging with heat sources, the optimal marker materials may vary in relation to the characteristics of the heat source.

Therefore, this study develops infrared markers that can be captured under heating and non-heating conditions in the medium wave infrared (MWIR) region. After four requirements for marker design are devised, the clearances of the thermal imaging of markers for 11 material combinations are evaluated under non-heating and heating conditions using an MWIR camera. Under heating conditions, the differences in marker-imaging characteristics between a hot-air heating using a heat gun and optical radiation heating using a halogen lamp are investigated.

We adopted the MWIR camera because the cooled InSb thermography camera will allow the users to capture images with high accuracy and speed. The MWIR camera has a variety of applications including material inspection and structural health monitoring [4,13,27,28,29], whereas the long wave infrared (LWIR) camera also has various applications [2,3,14,17]. Note that all the results of infrared images presented in this paper were captured with the MWIR camera (wavelength: 2–5.7 µm) and the markers may have different characteristics between the MWIR and LWIR regions, which will be revisited in the Discussion section.

In summary, the main contributions of our study are as follows.

1. We propose four requirements of infrared markers for engineering applications (e.g., device calibration and structural health monitoring), and investigate the appropriate combinations of materials based on these requirements.
2. We qualitatively and quantitatively compare the clearance of the thermal imaging of the markers under both non-heating and heating conditions in the MWIR region.
3. We evaluate how the difference in heat sources affects the imaging performance of the markers by comparing a hot-air heating with optical radiation heating.

## 2. Marker Design Requirements and Production

### 2.1. Design and Evaluation Requirements

This study proposes four requirements for marker design and evaluation as follows.

1. Available under both non-heating and heating conditions: the images are available under both conditions
2. Highly accurate production for imaging: the production of the markers is sufficiently accurate to capture clear images.
3. Compatible with both MWIR and visible cameras: the images can be captured using both cameras.
4. Easy to attach and detach: the markers should be easily attached and detached at the beginning and end of their utilization.

Requirement 1 indicates that the markers should be clearly captured under both non-heating and heating conditions. The target is often heated by a heat source under several thermal imaging, including active thermography methods for infrastructure inspection using a halogen lamp [30]. Thus, it is desirable that the markers be used without heating, as well as during and after heating. This study evaluates the performance of the markers under both non-heating (Experiment 1) and heating conditions (Experiment 2).

Requirement 2 states that the markers should be processed with an accuracy enough to capture clear images. The production accuracy of markers can affect the reliability of marker-based evaluation and calibration. For example, it is desirable for the markers to have clear contour lines for calibration, such that the patterns of a resolution chart can be calibrated. This study evaluates this type of accuracy from the obtained marker images.

Requirement 3 indicates that both MWIR and visible cameras should capture the marker. For multi-modal imaging, infrared and visible cameras are integrated, and compatible markers with both cameras enable simultaneous calibration and evaluation. This study compares the marker images that are captured by both MWIR and visible cameras in Experiment 1.

Requirement 4 refers to the easy attachability and detachability of markers in a laboratory or on-site. The rapid affixation and detachment of the markers are desirable for the improvement of evaluation tests and short time-efficient inspections. Note that methods using thermal paper [31] are irreversible, and methods using open holes on panels [17,18] are not versatile because they require depth. Thus, we adopt a sticker-type marker that can be easily installed.

In summary, this study evaluates the acquisition of clear images (Requirement 2) of a sticker-type marker (Requirement 4) using both MWIR and visible cameras (Requirement 3) under non-heating and heating conditions (Requirement 1).

### 2.2. Marker Production Procedures

**Figure 1:**
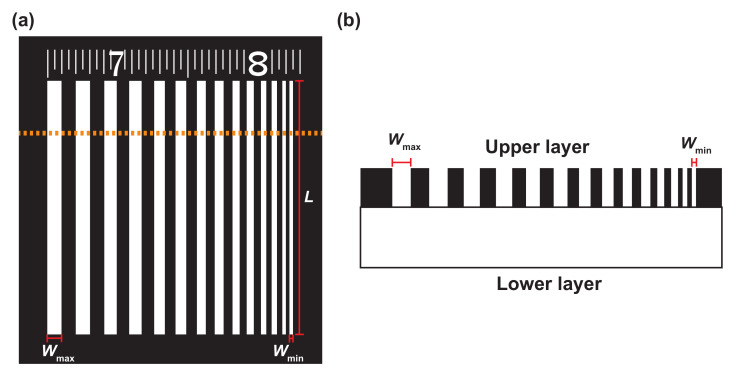
Marker used in this study: (**a**) basic structure; (**b**) cross-sectional view of the marker.

The basic structure of markers is shown in Figure 1a. The basic pattern comprises white lines of length *L* on a black background. The maximum width $W_{\max}$ to minimum width $W_{\min}$ had an arithmetic progression of 0.125 mm at every other line, containing 7 pairs of 14 lines. Markers with L=18.0 mm, $W_{\max}=1.0$ mm, and $W_{\min}=0.25$ mm were produced in Experiments 1 and 2.

Two types of materials were employed to create the sticker-type marker. Previous studies [15] used a metal–insulator combination of aluminum and polyvinyl tape, and the combination is often used as a material for calibration boards [6,13,14,15]. However, given the expected application of markers under both heating and non-heating conditions, it is necessary to investigate more wide-ranging materials. Our study compares 11 material combinations, as detailed in Table 1. Specifically, paper tape was added as a candidate insulator for marker materials because it is seal-like and can be easily combined with other materials. In addition to the aluminum used in the previous study, we also employed copper foil tape as the metal for marker materials. The abbreviations of each material are provided in Table 2. Cu and Al tape were used as the lower layers of markers (a)–(d) and (e)–(h), respectively, and the upper layers were made of BJP, WJP, PE-1 and PE-2 tape. Markers (a)–(h) were metal–insulator markers, with a metal on the lower layer and an insulator on the upper layer. Note that the insulators are easily cut with a laser cutter, which was used for our marker processing. Markers (i) and (j) were insulator–insulator markers with BJP and WJP. Marker (k) was printed on copier paper; it was used in a previous study to evaluate a motion-blur compensation system in the visible region [32].

As shown in Figure 1b, all markers, except for marker (k), were created by overlapping two seal-like materials and etching only the upper layer, such that the bottom layer was exposed at the bands as white lines (Figure 1a). These tapes were etched using a laser cutter (Universal Laser Systems, VLS3.50, spot size: 0.127 mm, processing accuracy: 500 dpi). The laser cutter used in this study melts the surface and removes it with assist gas. It is suited for the production of metal–insulator patterns because the insulators are etched more easily than metals. Insulator–insulator patterns can also be produced by regulating the irradiation intensity. Such markers have attachable/detachable tape with the adhesive surface of the lower layer.

### 2.3. Produced Marker Images and Qualitative Evaluation

**Figure 2:**
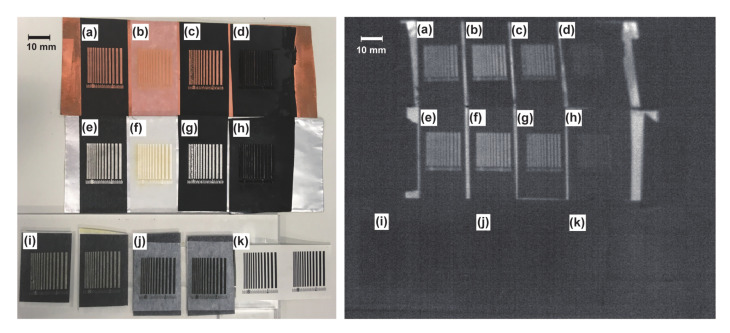
Images of produced markers captured by (**left**) visible camera (iPhone SE, pixel numbers: 1280 × 960) and (**right**) MWIR camera (InfRec H9000, pixel numbers: 640 × 512).

The marker production results are shown in Figure 2. Figure 2 (left) indicates visible camera images, and Figure 2 (right) indicates infrared camera images. The letters in the image correspond to the indices in Table 1. Two specimens are shown for markers (i), (j), and (k) to demonstrate the reproducibility of laser cutting and printing. Note that the quantitative evaluations of the production accuracy are discussed in Section 3. In this section, the qualitative observations of the produced markers are summarized.

#### 2.3.1. Comparisons of Upper-Layer Insulators

We compared markers (a)–(d) and (e)–(h), which had the same material on their bottom layers. The lines, having a $W_{\min}$ of 0.25 mm, were producible on markers (a), (b), (e), and (f), whereas the four lines having the lowest widths on markers (c) and (g) had uneven central lines. Additionally, markers (d) and (h) had insufficient production, such that the upper layer adhered to the lower one. The markers, using PE tape as an upper layer, showed production disturbances. Additionally, it was not possible to observe clear stripe patterns in the infrared images of markers (d) and (h), owing to insufficient production accuracy. These results indicate that markers combining PE tape and metal foil were insufficient for production via the laser cutter. However, the markers that combined Japanese paper tape and metal foil were accurately produced. Based on the infrared image shown in Figure 2 right, the comparison of metal–insulator markers (a)–(h) reveal that differences in marker clearance were developed according to the production accuracy of the visible images. Moreover, the comparison of markers (c) and (d) suggests that their production accuracy would be qualitatively different depending on the surface processing, although the main material of the upper layer was the same.

In summary, the production accuracy varied by material, and higher production accuracy was achieved for the combination of Japanese paper tape and metal foil in both visible and MWIR images.

#### 2.3.2. Comparisons of Lower-Layer Metals

We compared markers (a) and (e) as well as markers (b) and (f), having the same upper layer. The results indicated no qualitative difference was caused by using copper and aluminum for either visible or MWIR images. In other words, the difference in production accuracy caused by the selection of lower-layer metal materials was not a dominant factor.

#### 2.3.3. Comparisons of Metal–Insulator and Insulator–Insulator Markers

We compared metal–insulator markers (a)–(h) and insulator–insulator markers (i)–(k). Markers (a), (b), (e), (f), (i), and (j), with Japanese paper tape as the upper layer, were compared. No production disturbance was observed in any of these markers in the visible images. However, the number on the scale was smudged in the metal–insulator markers and was visible in the insulator–insulator markers. The production accuracy of the scale lines also indicates that the visible camera could capture insulator–insulator markers more clearly.

For MWIR images, metal–insulator markers (a)–(h) were captured, whereas clear marker images were not obtained for any of the insulator–insulator markers, (i)–(k). Note that this includes marker (k), which was used in a previous study [32]. These markers were difficult to use under non-heating conditions in the MWIR region. However, the presence of heat sources may affect the temperature difference between two materials of the marker, which will be verified in Experiment 2 of our study.

In summary, the comparison of the metal–insulator and insulator–insulator markers indicated that insulator–insulator markers were superior in terms of production accuracy in the visible region. In contrast, the insulator–insulator markers were unusable in the MWIR region. Hence, only the metal–insulator markers could be captured in the MWIR region clearly. However, note that bulk materials (e.g., stone and wood) were not included in our study, because they were not suitable for attachable seal-like markers.

#### 2.3.4. Production Reproducibility under Identical Conditions

We evaluated the reproducibility under identical productive conditions using markers (i) and (j). Qualitative differences were not observed, which indicates that laser-cutter-based production has sufficient reproducibility. Additionally, marker (k), composed of copier paper, was accurately reproduced.

## 3. Experiment 1: Marker Performance Evaluation under Non-Heating Conditions

This section presents the experimental procedures and results for the marker performance evaluation under non-heating conditions. Both visible and MWIR cameras were used to compare the characteristics of 11 markers under non-heating conditions.

### 3.1. Performance Evaluation Procedures

The 11 markers described in Table 1 were affixed to an iron plate perpendicular to the ground. The measurement system comprised the marker and camera. The mid-infrared camera (Nippon Avionics, InfRec H9000, pixel numbers: 640 × 512, spectrum: 2–5.7 µm) and visible camera (Ximea, MQ003MG-CM, pixel numbers: 648 × 488) were positioned at a distance of 500 mm and an angle of 0°, perpendicular to the iron plate. The imaging performance of the markers was evaluated as follows. First, a profile was created by taking the average of 10 lines on the marker at 10-pixel intervals for visible images and 5-pixel intervals for infrared images. Then, the maximum and minimum values in the profile were extracted, and the difference between the values was adopted as the evaluation value. Note that the maximum and minimum values, corresponding to the marker band, were extracted manually by adjusting the position of the marker image, owing to the noise that exists in the profile.

### 3.2. Results

**Figure 3:**
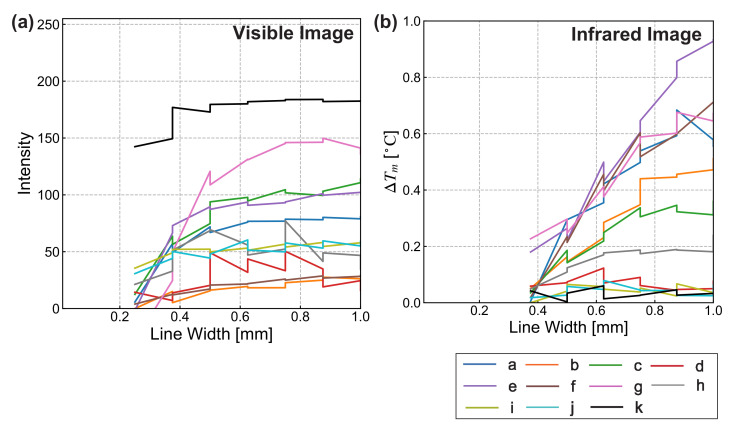
Marker performance under non-heating conditions: (**a**) intensity according to visible image; (**b**) temperature according to MWIR image.

An evaluation of the marker performance under non-heating conditions in the visible and MWIR region is shown in Figure 3 with the line width of marker bands on the horizontal axis and the difference between the maximum and minimum values of the profile on the vertical axis. This indicates the pixel-number difference of the 8-bit grayscale image in Figure 3a and the temperature difference estimated from the infrared camera in Figure 3b. The results of infrared images are presented up to the 11th band (0.375 mm), due to the influence of camera resolution.

We first compared the results for the visible images shown in Figure 3a. Quantitative observations indicated that marker (k) had the highest intensity values, followed by markers (a), (c), (e), and (g), comprising markers composed of BJP or PE tape. These were followed by insulator–insulator markers (i) and (j). Markers (d) and (h), having low production accuracy, had the lowest intensities. Additionally, markers (b) and (f), which contained markers made of WJP tape, had the lowest. The results for markers (b) and (f) were probably caused by the transparency of the WJP tape over the metal. In contrast, the BJP and PE tape had a high absorption coefficient in the visible spectrum. Thus, the intensity difference of their markers materials was higher.

Next, we compared the MWIR images shown in Figure 3b. Large temperature differences were obtained for markers (e)–(g), comprising aluminum tape with sufficient production accuracy. Markers (a)–(c) composed of copper tape had the next-highest differences. Markers (d) and (h), having a low production accuracy, and insulator–insulator markers (i)–(k) had the lowest values. In the MWIR images, the intensity difference depends on the emissivity in the MWIR region of the materials. From the experiment, the observed temperature difference of markers was larger when the combination of metals and insulators was adopted. Note that some results that show a monotonically increasing trend would be caused by the resolution of the MWIR camera.

## 4. Experiment 2: Marker Performance Evaluation under Heating Conditions

This section reports the procedures and results under heating conditions. The performances of the 11 markers were captured using the MWIR camera under heating and post-heating conditions.

### 4.1. Experimental Procedures

**Figure 4:**
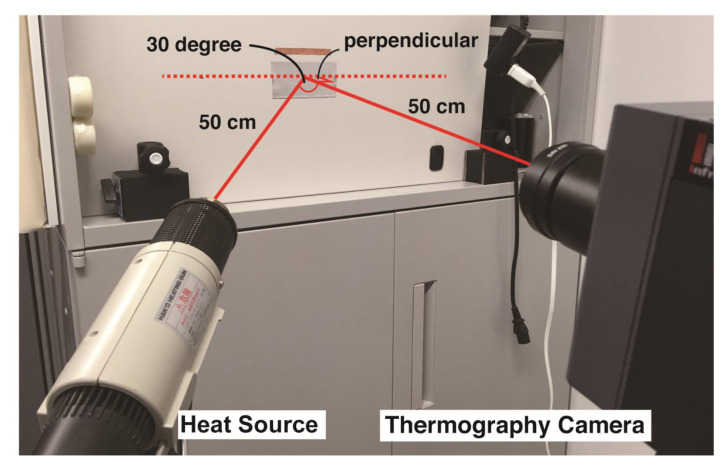
Photograph of experimental setup for measuring of heating characteristics.

The 11 markers were the same as in Experiment 1, and were affixed to an iron plate. The heating source was added to the setup of Experiment 1 at a position of 500 mm and an angle of 30° perpendicular to the board. The equipment is shown in Figure 4.

A halogen lamp (Victor, Video Light VL-P35, 300 W) and heat gun (HAKKO, Heating gun, No. 882, 1000 W) were used as heat sources, and an MWIR camera (Nippon Avionics, InfRec H9000, resolution: 0.025 ${}^{\circ }C$) was used for imaging. The average values of temperature difference between the minimum and maximum in the widest four bands of the markers were adopted for the evaluation. For recording the average performance of the marker imaging, heating was started 5 s after the beginning of the imaging by the MWIR camera. Heating was conducted for approximately 30 s to determine the heating characteristics of each marker. The camera continued to capture images for approximately 30 s even after the termination of the heating to verify the heat dissipation characteristics of the marker. In total, the MWIR camera captured each marker for 70 s. Each marker was attached to the same position on the iron plate. Note that the maximum and minimum values, corresponding to the marker band, were extracted manually by adjusting the position of the marker image, owing to the noise that exists in the profile.

### 4.2. Results

**Figure 5:**
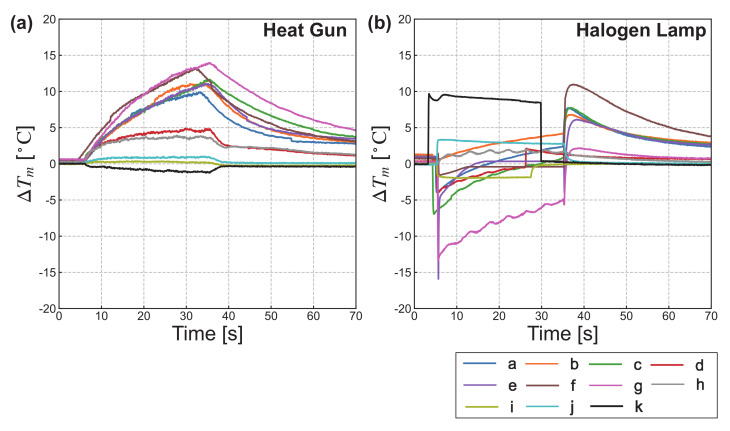
Results of temperature differences over time for (**a**) heat gun and (**b**) halogen lamp. Each color represents a different marker. The beginning and termination of the heating was manually switched.

The results are plotted in Figure 5 with the elapsed time from heating initiation on the horizontal axis and the temperature differences on the vertical axis. A different color represents each marker. Figure 5a,b show the experimental results obtained using the heat gun and halogen lamp, respectively. Note that the temperature difference of $\Delta T_{m}$ in Figure 5 does not indicate the difference from heating initiation $\Delta T_{t}$; rather, it indicates the difference between two materials for each marker.

First, we summarize the experimental results obtained when using the heat gun. Sharp changes immediately after heating initiation or termination were not observed. The smallest temperature differences were for the insulator–insulator markers (i)–(k), followed by markers (d) and (h), which had low production accuracy in the observation of Experiment 1. All other markers had clear temperature differences during and after heating.

Next, we summarize the experimental results obtained when using the halogen lamp. Unlike in the case of the heat-gun, abrupt changes were observed for each marker. At the beginning of heating, large differences were observed for markers (e), (g), and (k), followed by markers (c), (i), and (j). Markers (d) and (h) had the next-highest temperature differences. The lowest values were observed for markers (a), (b), and (f), comprising copper, aluminum, BJP, and WJP tape. The absolute temperature difference during heating increased in marker (b), and virtually no changes were observed in markers (e), (f), (i), and (j). All other markers showed decreasing tendencies. After heating, markers (d) and (h)–(k) had no differences, whereas all other markers had higher peaks and clear temperature differences. Additionally, all markers, except for markers (b), (f), and (h)–(k), crossed the zero point of $\Delta T_{m}\left(t\right)$. That is, there was a certain time during which they could not be temporarily captured during heating with a halogen lamp.

In summary, when heating using a halogen lamp, it is preferable to use insulator–insulator markers, although metal–insulator markers provide a clearer marker image during heating with a heat gun. Additionally, metal–insulator markers are preferable after heating with a heat source. A detailed evaluation based on the four requirements described in Section 2.1 is presented in Section 5.2. Note that these results were obtained by the MWIR cameras and may depend on the wavelength band of the camera. Details are given in the Discussion section.

## 5. Discussion

In this section, we describe the experimental results from the production and imaging characteristics of markers under both non-heating and heating conditions based on the thermal radiation theory (see in Appendix A). Then, we discuss these results based on the thermal radiation theory and rethink our marker-design requirements.

### 5.1. Marker Characteristics from Thermal Radiation Theory

Under the non-heating condition, considering Equation (A2), the reflected component in relation to the surrounding environment and the radiated component from the material reached the image sensor of the infrared camera. Although the specular component of reflectivity $\rho_{\mathrm{env}}$ is higher for metals and the emissivity ϵ is higher for insulators, the results of Experiment 1 suggest that the emissivity was a dominant factor. Additionally, the absence of emissivity difference between insulators caused inclearance of capturing the insulator–insulator markers. It should be noted that imaging performance of the marker may change in relation to the imaging angle due to the angular dependence of the specular reflective component and illumination emitting infrared lights. In an outdoor environment, the radiation from the ground and reflection of sunlight would also affect the marker clearance [5]. Considering the imaging performance of the metal–insulator markers, the images taken from an angle with many specular reflections would have more blurred markers than the one taken in our experiment.

Under the hot-air heating condition using a heat gun, considering Equation (A5), the temperature rise in the markers depends on the emissivity ϵ and the heat capacity *C*. Generally, both ϵ and *C* are high in insulators and have a trade-off relationship. Regarding the results of Experiment 2, the image clearance of the metal–insulator marker increased as the heating time elapsed under the condition, suggesting that the emissivity ϵ dominantly affected the temperature-rise rate. Conversely, because the rate of temperature rise between the white and black Japanese paper tape had no difference in this condition, the insulator–insulator marker did not appear as a clear image on the infrared camera, even when the heating time increased.

Under optical radiation heating using a halogen lamp, the characteristics of the markers differed, depending on the combination of metals and insulators. Particularly, several markers decreased their temperature differences during heating, whereas others increased their differences, owing to the presence of a reflection component ρ in addition to the radiation increase. There is a trade-off between the reflectivity and temperature-rise rate, depending on the combination of materials. In Experiment 2, the markers whose temperature difference decreased during heating were first dominated by the reflection component immediately after the beginning of heating, which was then replaced with the emissivity component as the heating time elapsed. Conversely, the markers whose temperature difference increased during heating lacked a trade-off between the reflection and absorption components, for which a clear marker image was obtained over time. Thus, before a marker is used, it is necessary to investigate whether there is a trade-off between the reflective and emissive components. Additionally, when the intensity of the halogen lamp becomes larger, the temporal dependence of the intensity of metal–insulator markers are qualitatively the same as that in our experiment, because both *W* and $W_{\rho }$ in Equation (A7) increased.

**Figure A1:**
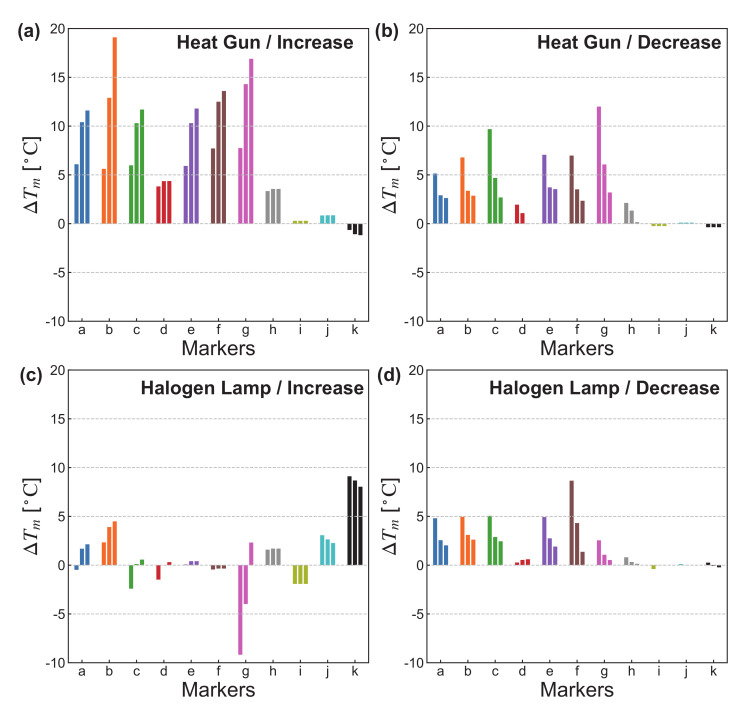
Fitting results for the data of Experiment 2 using (**a**,**b**) heat gun and (**c**,**d**) halogen lamp. The three bars shown in the same color are the temperature difference, starting from the left, 10, 30, and 60 s from the heating initiation in (**a**,**c**) and from the heating termination in (**b**,**d**).

In contrast, different experiment conditions from ours may cause the different imaging characteristics of each marker. For example, the camera angle would alter the intensity of the steep change that occurred immediately after the beginning of heating. This reflection components would be distinguished by a polarizer based on polarization theory as described in a recent research [33]. Furthermore, in the early times after the beginning of heating, the parameters of heat conduction, including the thermal conductivity, the mass density, and substrate thickness, would affect the temperature rise as a temporal function. The presence of the heat conduction terms may be the reason why the multi-exponential approximation provides a better fit in the fitting results of Figure A1 (See Appendix B for details of the fitting results under heating conditions).

The users should also be aware of the wavelength bands of capturing devices, especially whether the LWIR or MWIR camera is used. Under the non-heating condition, the optimal markers would not have a significant change between MWIR and LWIR. This is because metal–insulator markers are also used in the calibration of the LWIR camera [14,17], and the emissivity of metals tends to be low in the LWIR region. However, the users need to be careful in interpreting the results under the heating conditions because the spectral intensity of heat sources vary on the wavelength. In particular, under the optical radiation heating condition, the wavelength dependence of the spectral intensity may have a different effect on the reflection component $k_{\rho }\rho W_{\rho }\Theta \left(t\right)$ in Equation (A7).

### 5.2. Evaluation of Marker Selection

This section discusses marker suitability for each condition by reconsidering the marker requirements described in Section 2.1. This is reformulated as seven criteria, as follows, where the easy-to-detach requirement is omitted, because all markers are satisfied with this requirement, and the difference in heating sources is key to marker clearance.

1. Production accuracy
2. Clearance of visible-light imaging
3. Clearance of infrared imaging under the non-heating condition
4. Clearance of infrared imaging during heating under the hot air heating conditions
5. Clearance of infrared imaging after heating under the hot air heating conditions
6. Clearance of infrared imaging during heating under the optical radiation heating conditions
7. Clearance of infrared imaging after heating under the optical radiation heating conditions

Criterion 1 corresponds to Requirement 2, Criteria 2 and 3 correspond to Requirement 3, and Criteria 4 to 7 correspond to Requirement 1 in Section 2.1, respectively.

Here, we summarize experimental results in view of these criteria. For production accuracy and clearance of images using a visible camera (Criteria 1 and 2), all markers are usable, except for markers (d) and (h). However, for the clearance of MWIR imaging (Criterion 3), the MWIR camera could not image insulator–insulator markers (i)–(k) in addition to markers (d) and (h), which had low production accuracy. Marker performance during heating depended on the heating sources. Under the hot-air heating condition with a heat gun (Criteria 4 and 5), markers (a)–(c) and (e)–(g) were captured clearly both during and after heating. Conversely, under the optical radiation heating condition using a halogen lamp (Criteria 6 and 7), metal–insulator markers had insufficient clearance over a certain time, owing to the trade-off relationship between the reflection and absorption components. In contrast, insulator–insulator markers (i)–(k) were captured clearly. After heating, only the metal–insulator markers with sufficient production accuracy had sufficient clearance.

Based on this analysis, we recommend optimal materials of seal-like markers for infrared imaging in the MWIR region, as follows. Under the non-heating condition, metal–insulator markers, comprising BJP and copper or aluminum, i.e., markers (a) and (e), would be more suitable. These markers could also be used during or after heating, except for a specific time under the optical radiation heating condition. When these markers are used during heating with a halogen lamp, it is necessary to perform measurement or calibration while avoiding the unusable time. As alternative markers, the insulator–insulator markers would work effectively during heating with a halogen lamp. In particular, marker (k), as described in a previous study [32], has clear bands during heating with the halogen lamp, suggesting that marker (k) is most suitable under this condition. However, these markers cannot be used under non-heating and after-heating conditions. Note that the modulation transfer function (MTF) of the optical system would affect all markers in the same way. In other words, the same percentage reduction, in contrast, by the MTF would not have a significant effect on the order of the markers’ clearance we mentioned because the markers consisted of the same structure (arithmetic progression of 0.125 mm at every other line) with the different materials. The measurement wavelength of the camera may also affect the performance of the markers, as mentioned in the previous section.

The appropriate combination of marker materials we have discovered has potential applications for creating calibration and evaluation markers in inspection and imaging using the infrared camera. In particular, our findings would be applied to infrared inspection and imaging situations with a heat source. However, the proposed markers had the limitations that their applicability varied with heating times under the optical radiation heating condition, and the marker performance would depend on the angular dependence of reflective components. Further prospects include the development of a robust marker under optical heating conditions. Marker durability under environmental changes other than short-term heating should also be investigated to better understand environmental temperatures and indoor/outdoor properties. Another future work is to compare the accuracy of marker imaging in LWIR region during and after heating, based on the requirements and marker design proposed in this study.

## 6. Conclusions

This study investigated optimal material combinations for producing infrared markers under non-heating and heating conditions through two experiments in the MWIR region. After four requirements for marker design were proposed, 11 candidate markers were captured by visible and MWIR cameras to determine whether they could meet these requirements. Under non-heating conditions, the visible camera captured both metal–insulator and insulator–insulator markers, whereas the MWIR camera captured only metal–insulator ones. Markers composed of Japanese paper tape and metal foil were most suitable for MWIR imaging under non-heating conditions. Under heating conditions, the optimal markers depended on the heating source. Using a heat gun, which had no reflective component in the MWIR region, the temperature difference did not change suddenly by switching the heat source, and the metal–insulator markers were captured more clearly. The image became clearer as the heating time elapsed. Conversely, when using a halogen lamp, which had a reflective component in the infrared region, a steep temperature change was observed during heat-source switching, leading to results in which several metal–insulator markers were captured inaccurately at certain times. The marker composed of copier paper and black ink was most suitable during heating with a halogen lamp, whereas the metal–insulator markers were more suitable after heating.

## Figures and Tables

**Table 1:** Overview of investigated material combinations in each marker.

Index	a	b	c	d	e	f	g	h	i	j	k
**Upper**	BJP	WJP	PE-1	PE-2	BJP	WJP	PE-1	PE-2	BJP	WJP	Ink
**Lower**	Cu	Cu	Cu	Cu	Al	Al	Al	Al	WJP	BJP	Paper

**Table 2:** Abbreviations and manufacturing details of materials.

Abbreviation	Material	Manufacturer and Product Number
Cu	Copper foil (Metal)	Nitoms, copper foil tape J3170
Al	Aluminum foil (Metal)	Nitoms, thick aluminum tape, J3090
BJP	Black Japanese paper (Insulator)	Kamoi masking tape, Japanese paper adhesive tape 220 color
WJP	White Japanese paper (Insulator)	Monotaro, masking tape for architecture and painting
PE-1	Black polyethylene tape (Insulator)	Diatex, MT-08-BK-50MM
PE-2	Black polyethylene tape (Insulator)	Nitto Denko, PE corrosion-proof tape No. 55
Paper	Copier paper (Insulator)	Daio Paper, recycled plain copier paper
Ink	Toner (Insulator)	Canon, toner cartridge CRG-040HBLK

## Data Availability

Because the captured data comprise more than 300,000 images (over 300 images/s, for 1000 s), captured image data underlying the results presented in this paper are not publicly available at this time but may be obtained from the authors upon reasonable request.

## Appendix A. Thermal Radiation Theory of Material Heating

Here, we summarize a thermal radiation theory of material heating. A heat gun and halogen lamp are used as heat sources for hot-air heating and optical radiation heating, respectively. The differences in heating methods that affect several thermal components are discussed theoretically below.

### Appendix A.1. Kirchhoff’s Law and Energy Conservation

According to Kirchhoff’s law of thermal radiation, emissivity ϵ and absorptivity α are equivalent, i.e., ϵ=α. Here, ϵ is the ratio of emitted energy $I_{e}$ to the theoretical radiant energy of a black body $I_{\mathrm{ideal}}$, i.e., $\epsilon =I_{e}/I_{\mathrm{ideal}}$. Additionally, α is the ratio of absorbed energy $I_{a}$ to total radiant energy $I_{\mathrm{total}}$, i.e., $\alpha =I_{a}/I_{\mathrm{total}}$.

Given the law of thermal energy conversation regarding the material surface, the sum of reflectivity ρ, the transmissivity τ, and α is equal to one (ρ+τ+α=1). Here, ρ and τ are the ratios of the reflected energy $I_{r}$ and of the transmitted energy $I_{t}$ to $I_{\mathrm{total}}$, respectively.

When τ is zero, the following equation is obtained from Kirchhoff’s law and the law of energy conservation:

(A1) ρ=1−ϵ.

### Appendix A.2. Thermal Imaging under Non-Heating Conditions

When a thermography camera captures an image, both the emissive component (from the target) and reflective component (in relation to the infrared radiation present in the environment) arrive at the image sensors of the camera under non-heating conditions. This can be described as

(A2) $$\tilde{\epsilon }=\epsilon +\rho_{\mathrm{env}},$$

where $\tilde{\epsilon }$ is the total calculated emissivity that arrives at an infrared camera, ϵ is the emissivity of the target, and $\rho_{\mathrm{env}}=\rho \cdot I_{\mathrm{env}}/I_{\mathrm{total}}$ is the converted reflectivity that is related to the infrared radiation present in the environment. When two objects of the same temperature composed of different materials are observed, they are captured by the infrared camera at different temperatures, depending on the intensity of $\tilde{\epsilon }$. Typically, ϵ and the diffuse component of $\rho_{\mathrm{env}}$ are higher in insulators than in metals. However, the specular component of $\rho_{\mathrm{env}}$ is higher in metals.

### Appendix A.3. Thermal Imaging under Heating Conditions

This study addresses two heating conditions, namely, hot-air heating using a heat gun and optical radiation heating using a halogen lamp. These conditions have different characteristics from the perspective of thermal imaging.

Thermal imaging can be separated into two steps, i.e., the heating source increasing the temperature of the target, and thermal radiation detection using the infrared camera. Here, it is assumed that ϵ and α do not depend on temperature. For simplification, the temperature of the heat source $T_{s}$ is sufficiently higher than the material temperature T(t), and the first-order term of temperature difference $T\left(t\right)-T_{0}$ can approximate the heat emission from the target.

Thermal imaging under the hot-air heating condition should be considered for heat absorption and radiation. The emission from the heat source *W* is absorbed in proportion to the absorptivity α, and the heat is radiated in proportion to the emissivity ϵ. The temporal dependence of temperature *T* is

(A3) $$\frac{dT(t)}{dt}=\frac{\alpha W}{C}-k\epsilon \left(T\left(t\right)-T_{0}\right).$$

Here, *C* is the heat capacity, *k* is the proportional constant, and $T_{0}$ is the initial temperature at t=0. By solving the differential equation, the temperature is acquired as

(A4) $$T\left(t\right)=T_{0}+\frac{W}{kC}\left(1-e^{-k\epsilon t}\right).$$

From this relationship, the temperature difference is obtained as

(A5) $$\Delta T_{t}\left(t\right)=T-T_{0}=\frac{W}{kC}\left(1-e^{-k\epsilon t}\right).$$

Additionally, once the heating process is completed, the radiation term of Equation (A3) disappears. The temperature decrease is then described as

(A6) $$\Delta T_{t}\left(t\right)=\left(T_{c}-T_{0}\right)e^{-k\epsilon t},$$

where $T_{c}$ is the temperature immediately after termination of heating.

In contrast, thermal imaging under the optical radiation heating conditions should be considered for the reflective component of the heating source $W_{\rho }$, in addition to heat absorption and emission. The temperature difference from the initial temperature while heating is

(A7) $$\Delta T_{t}\left(t\right)=\frac{W}{kC}\left(1-e^{-k\epsilon t}\right)+k_{\rho }\rho W_{\rho }\Theta \left(t\right),$$

where ρ is reflectivity, Θ(t) is the Heaviside step function, and $k_{\rho }$ is the proportional constant. After heating, the expression becomes the same as Equation (A6). Note that the reflective component is predominant immediately after heating begins, whereas the contribution of the absorptive component becomes larger as heating continues; only the absorptive component is influential after heating.

## Appendix B. Fitting Results under Heating Condition

Here, a more detailed evaluation is performed by applying a fitting model to the data in Experiment 2. When heating with a heat gun, considering Equation (A5) and adding the constant caused by the offset for the difference of materials, we have

$$\begin{array}{c} \Delta T_{m}^{\mathrm{inc}}\left(t\right)=a_{i}\left(1-e^{-b_{i}\left(t-c_{i}\right)}\right)\Theta \left(c_{i}\right)+d_{i}\\ \Delta T_{m}^{\mathrm{dec}}\left(t\right)=\left\{a_{d}e^{-b_{d}\left(t-c_{d}\right)}+d_{d}e^{-e_{d}\left(t-c_{d}\right)}\right\}\Theta \left(c_{d}\right)+\left(a_{d}+d_{d}\right)\left(1-\Theta \left(c_{d}\right)\right)+f_{d}. \end{array}$$

where $\left(a_{i},b_{i},c_{i},d_{i}\right)$ and $\left(a_{d},\cdots ,f_{d}\right)$ are the fitting constant, and $\Delta T_{m}^{\mathrm{inc}}\left(t\right)$ and $\Delta T_{m}^{\mathrm{dec}}\left(t\right)$ denote the temperature difference between two marker materials during temperature increase and decrease, respectively. Additionally, when heating with a halogen lamp, considering Equation (A7), we obtain

$$\begin{array}{c} \Delta T_{m}^{\mathrm{inc}}\left(t\right)=\left\{a_{i}e^{-b_{i}\left(t-c_{i}\right)}+d_{i}e^{-b_{i}\left(t-c_{i}\right)}\right\}\Theta \left(c_{i}\right)+e_{i}\left(1-\Theta \left(c_{i}\right)\right)\\ \Delta T_{m}^{\mathrm{dec}}\left(t\right)=\left\{a_{d}e^{-b_{d}\left(t-c_{d}\right)}+d_{d}e^{-e_{d}\left(t-c_{d}\right)}\right\}\Theta \left(c_{d}\right)+\left(a_{d}+d_{d}\right)\left(1-\Theta \left(c_{d}\right)\right)+f_{d}. \end{array}$$

For the fitting, $\Delta T_{\mathrm{inc}}\left(t\right)$ was estimated using 0–24.5 s data every 0.07 s under all conditions, whereas $\Delta T_{\mathrm{dec}}\left(t\right)$ was estimated with 35–70 s data at 0.07 s intervals under all conditions.

Figure A1 shows the fitting results for the data of Experiment 2 with the markers on the horizontal axis and the temperature difference between two materials on the vertical axis. The three bars shown in the same color denote data, starting from the left, 10, 30, and 60 s from heating initiation in Figure A1a,c and from the heating termination in Figure A1b,d. Figure A1a,b denote the results obtained using a heat gun, and Figure A1c,d denote those obtained using a halogen lamp.

Under the heat-gun condition, all markers, except for marker (i), were captured during heating, and the insulator–insulator markers could not be captured afterwards. Markers (d) and (h), which had production inaccuracies, could not be imaged at 60 s after heating. These results suggest that the metal–insulator markers should be used when heating with a heat gun. Under the halogen-lamp condition, all metal–insulator markers, except for markers (b) and (f), crossed the zero point of their temperature difference, whereas all insulator–insulator markers maintained the signature of temperature difference. Thus, using the insulator–insulator markers is recommended during heating. Conversely, after heating, all insulator–insulator markers had low temperature differences, indicating that the insulator–insulator markers should be used only during halogen-lamp heating. Previous studies using markers for camera calibration [11,12] did not investigate the effect of heat sources on the printed markers, especially temporal dependence of the markers contrast. From the experimental results, we suggest that the printed markers can be clearly captured immediately after the beginning of heating when a halogen lamp, which has a reflective component in the infrared region, is utilized as the heating source.
